# Examining the Role of Responsibility and Belief in a Just World in the Relationship between Parental Rejection and Adolescents’ Gratitude

**DOI:** 10.3390/bs13040305

**Published:** 2023-04-03

**Authors:** Jingwen Xing, Xiaofeng Xu, Xingchao Wang

**Affiliations:** 1 School of Primary Education, Shanghai Normal University Tianhua College, Shanghai 201815, China; wxing@chapman.edu; 2 School of Health, Shanghai Normal University Tianhua College, Shanghai 201815, China; xxf1132@sthu.edu.cn; 3 School of Education Science, Shanxi University, Taiyuan 237016, China

**Keywords:** parental rejection, gratitude, responsibility, belief in a just world, high school students

## Abstract

Parenting is an important factor affecting teenagers’ gratitude, but few studies have deeply examined the impact of specific parenting behavior on teenagers’ gratitude. In this study, 357 high school students were tested by questionnaires to examine the mechanism of parental rejection on adolescents’ gratitude. Results showed that (1) parental rejection significantly and negatively predicted adolescents’ gratitude, and that (2) after controlling for gender and age, parental rejection would indirectly impact adolescents’ gratitude through responsibility and belief in a just world, respectively. These results suggested that responsibility and belief in a just world were important factors in reducing the negative effect of parental rejection on teenagers’ gratitude.

## 1. Introduction

Gratitude is a traditional virtue of the Chinese people and is the way of being human. In ancient times, there were such classical stories as “Di shui zhi en, yong quan xiang bao”, which means if someone offers us a drop of water when we are in need, we will repay their kindness with a spring. The Chinese character “Gratitude” originated from the classics “San Guo Zhi-Wu Zhi-Luo Tong Zhuan”, which said: “In ancient China, during the Three Kingdoms Period, the Emperor of Kingdom Wu, Sun Quan, was persuaded by Luo Tong (a general of Wu) to employ the following actions on days of feasting to reward his men: he would invite each of them to meet him individually, ask about their lives, give them intimate affection, induce them to talk to him, and examine their interests so that they would be grateful and want to return the favor”. Currently, gratitude is an emotional trait of one individual who recognizes the kindness or help of the benefactor and tries to be rewarded [1,2]. Previous studies on gratitude focused more on college students [3,4] or special groups [5,6], and less on teenagers in general [7]. Moreover, adolescence is a critical period for cultivating and developing an individual’s gratitude [8]. Therefore, elucidating the risk and protective factors may not only promote gratitude and related behaviors in adolescents but also contribute to their overall healthy development.

According to the ecosystem theory, the family acts as a microsystem of individual growth, and the development of gratitude is influenced by family factors [9]. As the internal environment of the family, parenting style has a long-term and profound impact on the children’s growth and social adaptation [7]. Previous studies have shown that different parenting styles generated different adaptive outcomes. Positive parenting produced good adaptation, which would fill individuals with hope and gratitude for life [10], while negative parenting hindered individual social adaptation [11]. Parental rejection is typical of negative parenting, predicting individual psychological and behavioral problems [11,12,13] and even, in severe cases, causing individual suicide [14]. Meanwhile, parents’ rejection can easily provoke children’s resentment towards their parents; this resentment may then be migrated to their social activities and consequently may render children’s hostility and aggression towards others [15]. Research also identified parental rejection as a negative factor affecting medical students’ gratitude [16]. It follows that parental rejection may be an important risk factor for youths’ gratitude. Therefore, we assumed that *parental rejection would negatively predict adolescents’ gratitude.*

Some studies demonstrated that parenting could influence adolescents’ gratitude directly and could also play a role through some important mediating roles (i.e., Zhao et al., 2018). In this study, we considered responsibility as a possible mediating role. Responsibility is an important personality trait of a person who consciously does the job well, and it is a psychological quality including emotion [17]. Literature documented that family had the greatest impact on the development of children’s sense of responsibility [18] while negative parenting practices (e.g., refusing to deny) hindered this development [19]. In addition, responsibility could positively predict adolescent gratitude. Responsibility is one of the mediating variables between parent–child conflict and gratitude [20]. As an important part of parent–child conflict, *responsibility could be assumed to play a mediating role between parents’ rejection and adolescents’ gratitude.*

Moreover, the belief in a just world, as a cognitive factor, refers to people who believe that they live in a fair world where they feel valued and self-sufficient [21] and that the world is stable and orderly, so that behavioral outcomes are predictable [22]. Belief in a just world helps people build a sense of control over the world, makes individuals more willing to follow the social norms, motivates them to pursue the long-term goals [23], and plays an important role in adapting individuals to complex social environments [24]. Belief in a just world can be divided into personal belief in a just world and general belief in a just world [25,26]. Belief in a just world is significantly associated with positive emotions, which are conducive to individual mental health [22]. According to the ecosystem theory, the parenting style, as a microsystem, will affect an individual’s belief in a just world and then affect their development in psychology and behavior. Studies showed that parental emotional warmth was a positive predictor [7,27,28], while negative parenting styles was a negative predictor of personal belief in a just world [29]. Moreover, Dalbert and Radant argued that different parenting styles would have different effects on the individual’s belief in a fair world. A healthy family climate and positive interactions between parents and children would constitute an ecosystem of fairness and equality, treating teenagers as independent individuals and improving the teenagers’ belief in the personal just world [29]. At the same time, some scholars pointed out that the more individuals believed that the world was just, the more prosocial behaviors there would be [30] and the higher level of gratitude [31]. People who believe in a fair world tend to believe that giving should be rewarded, and those who receive favors should be grateful to others [32]. Recent studies suggested that belief in a just world played a mediating role in the impact of parents’ emotional warmth on adolescents’ gratitude [7]. In addition, as an important part of negative parenting, parental rejection possibly is associated with gratitude through belief in a just world. Thus, we hypothesized that *belief in a just world would play a mediating role between parental rejection and adolescents’ gratitude*.

Furthermore, the current study aims to explore the effect of parental rejection on adolescents’ gratitude and the mediating role of belief in a just world and responsibility between parental rejection and adolescents’ gratitude. Zhao et al. [7] considered belief in a just world and responsibility as parallel mediating variables. The reasons are as follows: (1) belief in a just world is a cognitive concept and responsibility is the emotional component of the individual factors [33]. (2) The two concepts are framed by two different theories, namely the social exchange theory and reciprocal determinism respectively [8]. Taking the two mediators as parallel variables helps to explore the influence of parenting styles on teenagers’ gratitude more comprehensively from different perspectives. (3) Both theories are very important for the growth and development of adolescents. The current study hypothesized that *responsibility and belief in a just world were parallel mediators in the relationship between parental rejection and adolescents’ gratitude*.

**Figure 1:**
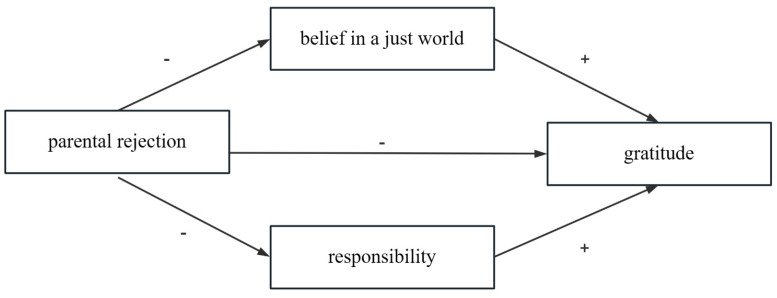
The conceptual mediating model.

In sum, the present research, based on the social exchange theory and reciprocal determinism, aims to investigate the relationship between parental rejection and gratitude and the mediating role of responsibility and belief in a just world between them. The conceptual model is shown in Figure 1.

## 2. Materials and Methods

### 2.1. Participants

Using convenient sampling, 420 questionnaires and 352 valid questionnaires were distributed in four middle schools in Shanghai, China. The mean age of the participants was 15.84 years (*SD* = 0.686), with an age range of 14 to 21 years. Among them, 112 (31.8%) were male, and 240 (68.2%) were female. The study was approved by the Institutional Review Board (IRB) of the first author’s affiliation, and the consent form was obtained before the researchers conducted the survey.

### 2.2. Measurements

#### 2.2.1. Parental Rejection (Parents Rejection Scale)

The rejection subscale in the simple parenting questionnaire was compiled by Jiang et al. [34], with a total of 6 items, such as “Father/Mother often loses their temper with me without me knowing the reason”. The scale was scored with 4 points, from 1 (never) to 4 (always). Higher scores indicate stronger parenting rejection to their children. The Cronbach’s *α* coefficient of the parental rejection subscale in this study was 0.895.

#### 2.2.2. Gratitude Questionnaire (The Gratitude Questionnaire-6, GQ-6)

The gratitude questionnaire prepared by McCulloch et al. [1] was adopted, which was revised by Yu et al. [35] and included six items: for example, “there are too many things to appreciate in life”. The subjects were required to evaluate according to their own current situation. The scale is scored with 7 points, from 1 (completely disagree) to 7 (fully agree), and the third and sixth questions are scored in the reverse direction: the higher the score, the stronger the tendency to be grateful. The Cronbach’s *α* coefficient of GQ-6 in this study was 0.771.

#### 2.2.3. Responsibility Questionnaire (Responsibility Questionnaire)

The overall responsibility questionnaire of middle school students was compiled by Tan Xiaohong [17] including a total of 5 items, such as “I am a responsible person”. The questionnaire used a 5-point score method, from 1 (very inconsistent) to 5 (very consistent). Higher scores indicate greater responsibility. In the current study, the Cronbach’s *α* coefficient of the overall Responsibility Questionnaire was 0.705.

#### 2.2.4. Belief in a Just World Scale

This scale, developed by Kay and Jost [36], includes nine items, such as “Overall, this is a fair world”, and others. The scale is scored at 7 points, from 1 (completely disagree) to 7 (fully agree), with the second, sixth, and ninth questions being scored in reverse. Higher scores indicate stronger fair-world beliefs. The Cronbach’s *α* coefficient of this scale in this study was 0.69.

### 2.3. Data Analysis

We used SPSS 25.0 to conduct the data analysis on descriptive analysis and correlation analysis. The mediating model test was employed by the SPSS PROCESS macro (http://www.afhayes.com, accessed on 1 March 2020) prepared by Hayes (2012). The Bias-correlated percentile Bootstrap method recommended by Fang, Zhang, and Qiu (2012) was used to evaluate the significance. If the Bootstrap 95% confidence interval did not include 0, then the effect was significant.

### 2.4. Common Method Bias Test

According to Zhou and Long [37], using confirmatory factor analysis, all items in the parenting style (parental rejection), gratitude, responsibility, and fair belief questionnaires were taken as explicit variables, and the number of common factors was set as 1. The results showed the fitting index as follows: *χ*^2^/*df* = 3.38, RMSEA = 0.08, NFI = 0.665, GFI = 0.801, CFI = 0.736. This indicates that there is no serious issue of common method bias in the present study.

## 3. Results

### 3.1. Descriptive and Correlational Analysis

The results showed significant positive associations between gratitude, responsibility, belief in a just world, and parental rejection (see Table 1).

### 3.2. Testing for the Proposed Mediation Model

After controlling for sex, age, and grade, the results of the mediation effects showed a significant negative prediction of gratitude (*β* = −0.123, *p* < 0.05) and gratitude (*β* = 0.265, *p* < 0.001; *β* = 0.369, *p* < 0.001) (see Table 2).

Analysis of the mediation effect of belief in a just world and responsibility between parental rejection and gratitude showed that the indirect effect of belief in a just world in the effect of parental rejection on gratitude was −0.047, and its Bootstrap 95% confidence interval did not include 0, indicating that the mediation effect of belief in a just world between parental rejection and gratitude was significant. The indirect effect of responsibility in parental rejection on gratitude was −0.042, and its Bootstrap 95% confidence interval did not contain 0, also indicating a significant effect of responsibility between parental rejection and gratitude.

**Figure 2:**
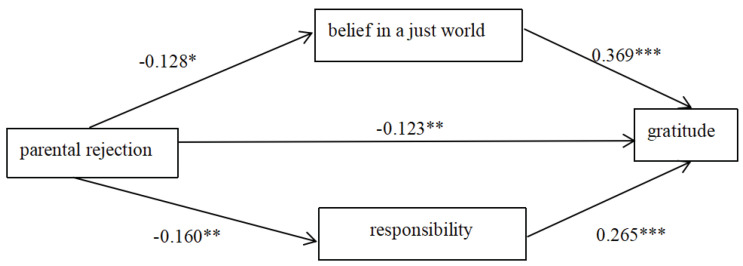
The mediating role of responsibility and belief in a just world. * *p* < 0.05, ** *p* < 0.01, *** *p* < 0.001.

In addition, the difference between mediation effect of belief in a just world and responsibility was 0.005, and its Bootstrap 95% confidence interval did not include 0, indicating that the effect of belief in a just world is significantly greater than responsibility in the effect of parental emotional warmth on gratitude (see Table 3). The relationships among main variables are showed in Figure 2.

## 4. Discussion

This study constructed a parallel mediating model to examine the effect of a specific negative parenting behavior—parental rejection—on adolescents’ gratitude. The results showed that parental rejection would negatively predict adolescents’ gratitude and could also predict adolescents’ gratitude through the mediating role of responsibility and belief in a just world.

### 4.1. Relationship between Parental Rejection and Adolescents’ Gratitude

According to the ecosystem theory [9], parenting, as an important part of the microsystem, has a profound impact on the development of teenagers. This study selected an important risk factor of negative parenting (parental rejection), and showed that parental rejection could be a significant and negative factor of adolescents’ gratitude. This result is consistent with previous research revealing that the higher the parental rejection, the lower the level of gratitude in adolescents [16]. Parental rejection is supposed to be a negative parenting style, which will jeopardize the individual’s social adaptation and impede the development of gratitude in young people. Therefore, parents may reduce or even avoid frequent rejection and instead adopt more positive parenting technique to nurture gratitude in adolescents..

### 4.2. The Mediating Role of Responsibility and Belief in a Just World

Research indicated that parental rejection not only directly influenced adolescents’ gratitude, but also indirectly affected gratitude in such a group through a mediator of responsibility. Some scholars proposed that teenagers who grew up in a family maintaining a negative parenting style (e.g., parental rejection), would perceive a weaker sense of responsibility and then show less gratitude behavior [19]. This is because among all the relevant factors family has the most profound impact on the development of sense of responsibility in adolescents [18]. Parental rejection will make their children indifferent to themselves or others, leading to a lack of responsibility as a result [38]. According to the theory of social exchange, when individuals accept the favor of others, the benefactor reward is a kind of responsibility. People with higher levels of responsibility are more likely to conform to the social norms, and have a tendency to exhibit gratitude behaviors or become benefactors. Meanwhile, people who lack responsibility will show less gratitude [1]. It can be seen that responsibility is a key protective factor and can reduce the negative impact of parental rejection on teenagers’ gratitude. Therefore, enhancing the sense of responsibility can not only foster gratitude in adolescents, but also effectively reduce the negative impact of negative parenting on young people’s gratitude.

Furthermore, this study found that belief in a just world played a mediating role in the relationship between parental rejection and adolescents’ gratitude. Here belief in a just world means that teenagers perceive the justice of the world. The higher their score on parental rejection, the less unfair their environment was and the less likely they were to demonstrate gratitude. This result is consistent with previous studies [29,31]. This may be due to a negative family atmosphere caused by parental indifference and rejection, in which individuals feel that they are not being treated fairly so that it is difficult to develop their fair beliefs about the world [29]. In addition, according to the principle of social reciprocity, the stronger the individual’s faith in the fair world, the more likely one is to express gratitude in social interactions [30]. Therefore, belief in a just world is a protective factor in the relationship between parental rejection and teenagers’ gratitude. These findings indicated that belief in a just world could weaken the negative impact of parental rejection on adolescents’ gratitude by improving the level of their fair world belief.

Finally, this study validated the parallel mediating role of responsibility and belief in a just world between parental rejection and adolescents’ gratitude through a dual-path model. That is, parental rejection could impact adolescents’ gratitude through both the cognitive path (fair world belief) and the emotional path (responsibility). Moreover, the study found that the effect of the cognitive pathway was significantly greater than that of the emotional pathway. Adolescence is an important transitional period for cognitive and emotional development [39]. Emotional development lags behind in contrast to the rapid development of cognitive ability and speedy physical maturity. Additionally, school education focuses more on the development of students’ academic performance so that their cognitive ability has a better chance to be trained. The findings in this study may broaden the understanding of the relationship between negative parenting and gratitude, enrich the theory of gratitude, and provide a new idea for nurturing gratitude in students.

## 5. Limitations, Future Directions, and Strength

The present study has the following limitations. First, this study was cross-sectionally designed and could not conclude a causal relationship between parental rejection and adolescents’ gratitude. In the future, longitudinal designs could be used to explore the developmental trajectory of the impact of parental rejection on adolescents’ gratitude. Secondly, this study only considered the mediating role of responsibility and belief in a just world, other mediating and moderating variables could also be investigated to further explore the mechanisms of parenting influence on adolescents’ gratitude. Finally, the current research only focused on individual and family factors; Future research could examine more social factors such as community, school, and society, as well as their impact on adolescents’ gratitude. Nevertheless, despite these limitations, this study provided a unique sample including diverse cultures of parenting, compared to the bulk of gratitude research conducted in the West which is more individualistic and capitalistic. The diversity of data constitutes an important and unique strength of the current research that should not be underestimated. Future study on multicultural background groups may warrant more attention.

## 6. Conclusions

The current research examined the association between parental rejection and adolescents’ gratitude and their mediating roles of belief in a just world and responsibility. The results showed that first, parental rejection significantly negatively predicted the adolescents’ gratitude; second, after controlling for gender, age, and grade, parental rejection could influence the adolescents’ gratitude through responsibility and belief in a just world. The findings from this study have implications not only for future research but for policy and practice. Government or policymakers may provide more parent education programs which could facilitate parents’ self-growth and increase their capacity for nurturing and nourishing the next generation.

## Figures and Tables

**Table 1:** Descriptive statistics and correlation analysis of variable.

	*M (SD)*	1	2	3	4
1 gratitude	5.13 (1.02)	1			
2 responsibility	3.97 (0.61)	0.402 **	1		
3 belief in a just world	4.58 (0.76)	0.476 **	0.325 **	1	
4 parental rejection	1.44 (0.49)	−0.218 **	−0.167 **	−0.14 **	1

** *p* < 0.01.

**Table 2:** Regression Analysis.

Model		Fit index in Total		Significance of Regression Coefficient	
Outcome Variables	Predictive Variable	*R^2^*	*F*	*β*	*t*
responsibility	gender	0.031	3.740 *	0.005	0.099
	age			0.058	1.079
	parental rejection			−0.160	−2.996 **
belief in a just world Scale	gender	0.027	3.208 *	0.087	1.614
	age			0.026	0.488
	parental rejection			−0.128	−2.391 *
gratitude	gender	0.315	31.862 ***	0.050	1.100
	age			−0.048	−1.059
	belief in a just world			0.369	7.786 ***
	responsibility			0.265	5.571 ***
	parental rejection			−0.123	−2.680 **

* *p <* 0.05; ** *p* < 0.01; *** *p* < 0.001. All variables were standardized when entered into the regression models.

**Table 3:** Analysis of the mediation effects of responsibility and belief in a just world in parental rejection influencing gratitude.

	Value of Indirect Effect	Boot SE	95%Boot LCI	95% Boot UCI	Relative Mediation Effect
total indirect effect	−0.094	0.033	−0.161	−0.033	73.06%
belief in a just world	−0.050	0.021	−0.095	−0.011	38.51%
responsibility	−0.045	0.021	−0.089	−0.008	34.55%
belief in a just world–responsibility	−0.005	0.027	−0.061	0.047	3.96%

## Data Availability

The data presented in this study are available on request from the first author. The data are not publicly available due to privacy or ethical restrictions.
